# New-onset ventricular arrhythmias post radiofrequency catheter ablation for atrial fibrillation

**DOI:** 10.1097/MD.0000000000004648

**Published:** 2016-09-09

**Authors:** Lingmin Wu, Yanlai Lu, Yan Yao, Lihui Zheng, Gang Chen, Ligang Ding, Bingbo Hou, Yu Qiao, Wei Sun, Shu Zhang

**Affiliations:** a State Key Laboratory of Cardiovascular Disease, Fuwai Hospital, National Center for Cardiovascular Diseases, Chinese Academy of Medical Sciences and Peking Union Medical College, Beijing; b Department of Immunology, Nanjing Medical University, Jiangsu, People's Republic of China.

**Keywords:** atrial fibrillation, inflammation, radiofrequency catheter ablation, ventricular arrhythmia

## Abstract

As a new complication, new-onset ventricular arrhythmias (VAs) post atrial fibrillation (AF) ablation have not been well defined. This prospective study aimed to describe the details of new-onset VAs post AF ablation in a large study cohort.

One thousand fifty-three consecutive patients who underwent the first radiofrequency catheter ablation for AF were enrolled. All patients had no evidence of pre-ablation VAs. New-onset VAs were defined as new-onset ventricular tachycardia (VT) or premature ventricular contractions (PVC) ≥1000/24 h within 1 month post ablation.

There were 46 patients (4.4%) who had 62 different new-onset VAs, among whom 42 were PVC alone, and 4 were PVC coexisting with nonsustained VT. Multivariate analysis showed that increased serum leukocyte counts ≥50% post ablation were independently associated with new-onset VAs (OR: 1.9; 95% CI: 1.0–3.5; *P* = 0.043). The median number of PVC was 3161 (1001–27,407) times/24 h. Outflow tract VAs were recorded in 35 (76.1%) patients. No significant differences were found in origin of VAs (*P* = 0.187). VAs disappeared without any treatment in 6 patients (13.0%). No VAs-related adverse cardiac event occurred.

The study revealed a noticeable prevalence but relatively benign prognosis of new-onset VAs post AF ablation. Increased serum leukocyte counts ≥50% post ablation appeared to be associated with new-onset VAs, implying that inflammatory response caused by ablation might be the mechanism.

## 1 Introduction

Radiofrequency catheter ablation (RFCA) for atrial fibrillation (AF) is increasingly used.^[1]^ Extensive ablation strategies including circumferential pulmonary vein isolation (CPVI), multilinear ablation, and complex fractionated atrial electrograms elimination are usually performed to improve the outcome.^[2–5]^ However, extensive ablation may lead to more complications. Patel et al^[6]^ first reported the new-onset outflow tract (OT) ventricular premature depolarization post RFCA in 53 AF patients. However, the small size of the study cohort limited their conclusion. In this prospective study, we aimed to describe the details of new-onset ventricular arrhythmias (VAs) post AF ablation in a large cohort, and to examine clinical variables associated with new-onset VAs.

## 2 Methods

### 2.1 Study population

The study enrolled consecutive patients who underwent the first RFCA for AF at our institution between January 2010 and July 2014. All patients failed to respond to antiarrhythmic drugs for rhythm control. The exclusion criteria included: patients had clinical history of VAs or structural heart diseases; patients had previous AF ablation history; patients had diseases which could affect the inflammatory state including pericarditis, myocarditis, connective tissue diseases, chronic inflammatory disease, infectious diseases, hepatic and renal dysfunction; patients had procedure-related cardiac tamponade or myocardial infarction. Paroxysmal AF was defined as AF that terminated spontaneously or with intervention within 7 days of onset, persistent AF was defined as continuous AF sustained beyond 7 days.^[1]^ The study was approved by the local ethical research committee. Informed consent was obtained from all patients.

### 2.2 Electrophysiological study and ablation procedure

All patients underwent 24-h 12-lead Holter and 48-h telemetry monitoring within 1 month before ablation. The procedure was performed under conscious sedation with an intravenous injection of midazolam and flurbiprofen. Three-dimensional geometry of the left atrium (LA) and pulmonary veins were constructed under the guidance of the Ensite NavX (St. Jude Medical, St. Paul, MN) or electroanatomical mapping system (CARTO, Biosense Webster Inc., Diamond Bar, CA).

All patients underwent CPVI, CPVI in combination with linear ablation, or pure linear ablation. The strategy of linear ablation has been previously reported.^[3,4]^ A 4-mm or 3.5-mm irrigated-tip ablation catheter (IBI, St. Jude Medical, Irvine, CA or Thermo Cool Navi-Star, Biosense Webster Inc., Diamond Bar, CA), or an 8-mm-tip ablation catheter (Bard Electrophysiology, Lowell or St. Jude Medical, Irvine) was used. For irrigated-tip catheters, radiofrequency lesions were made at a target temperature of 43°C and maximum output of 40 W. For the 8-mm-tip catheters, settings of temperature ≤58°C and power ≤60 W were used.

### 2.3 Data collection and follow-up

All patients were monitored for 48 h in hospital after ablation. Previously ineffective antiarrhythmic drugs were continued for 3 months. Routine blood examinations made including level of serum N-terminal pro B-type natriuretic peptide (NT-proBNP) and C-reactive protein before ablation. Post-ablation routine blood tests were repeated within 24 h. Follow-up including echocardiography, electrocardiogram, and 24-h 12-lead Holter were obtained at 1, 3, 6, and 12 months after ablation. In addition, electrocardiograms were recorded at the times of symptoms, and 12-lead Holters were also repeated in patients without recorded VAs.

New-onset VAs were defined as new-onset ventricular tachycardia (VT) or premature ventricular contraction (PVC) ≥1000/24 h within 1 month post ablation. Nonsustained VT was defined as runs of beats arising from the ventricles with duration between 3 beats and 30 s and with cycle length of <600 ms.^[7]^ OT VAs were defined as VT/PVC with a QRS complex >120 ms, positive deflection in inferior wall leads. VAs originating from right ventricular outflow tract (RVOT) were defined as an inferior axis in the frontal plane and left bundle branch block configuration with precordial R/S transition after lead V_3_ or R/S transition later than sinus rhythm, or R/S transition <0.6 in lead V_2._ Left ventricular outflow tract (LVOT) VAs were defined as either a right bundle branch block/inferior axis or a left bundle branch block/inferior axis with a precordial R/S-wave transition before lead V_3_ or R/S transition >0.6 in lead V_2_.^[8–11]^

### 2.4 Statistical analysis

Statistical analyses were performed using SPSS 19.0 software (SPSS Inc., Chicago, IL). Comparisons of continuous variables were performed with the Student *t*-test or Wilcoxon test and categorical variables with χ^2^ analysis. For the multivariate logistic regression analysis, the continuous variables were appropriately transformed where required to render them normally distributed. The baseline variables including age, sex, AF classification, AF history, occurrence of comorbidities, echocardiography parameters, serum NT-proBNP level, serum leukocyte counts, serum C-reactive level, and whether heart rate (HR) increased 1 month post ablation were analyzed to evaluate their association with the presence of new-onset VAs. All tests were two-tailed and a statistical significance was established at a *P* < 0.05.

## 3 Results

### 3.1 Patient characteristics

From January 2010 to July 2014, 1574 patients underwent RFCA for AF at our institution and 1053 patients (797 men and 256 women) who met the inclusion criteria were enrolled. Baseline characteristics are presented in Table 1.

**Table 1:**
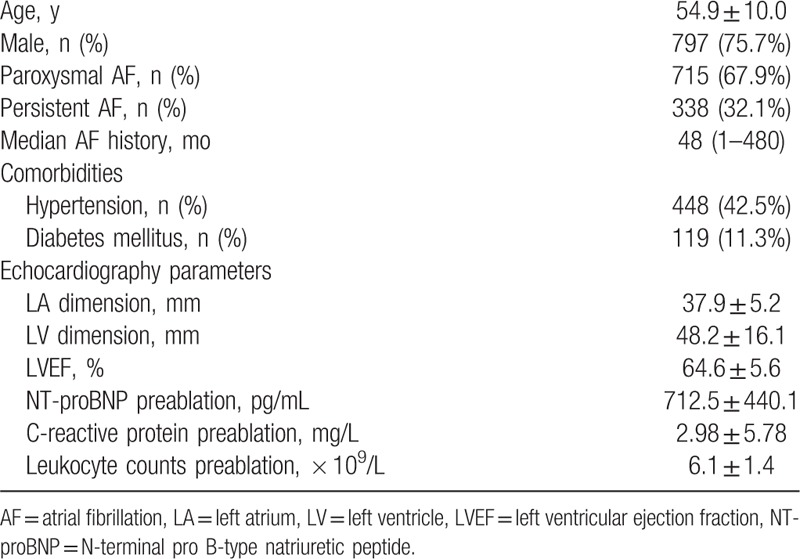
Baseline characteristics of the study cohort (n = 1053).

### 3.2 Prevalence and predictor of new-onset VAs

There were 46 patients (4.4%) who showed new-onset VAs post ablation, among whom 42 showed frequent PVC alone, and 4 showed frequent PVC coexisting with nonsustained monomorphic VT. A total of 62 different VAs were recorded, the mean number of VA morphologies in each patient was 1.3. The number of VA morphology was 1 in 33 (71.8%) patients, 2 in 10 (21.7%), and 3 in 3 (6.5%).

Comparison of characteristics between patients with and without new-onset VAs is shown in Table 2. Univariate analysis showed significant statistical difference in post-ablation serum leukocyte counts (*P* = 0.026). Multivariate analysis showed that an increase of ≥50% in post-ablation serum leukocyte counts was independently associated with new-onset VAs (OR: 1.9; 95% CI: 1.0–3.5; *P* = 0.043).

**Table 2:**
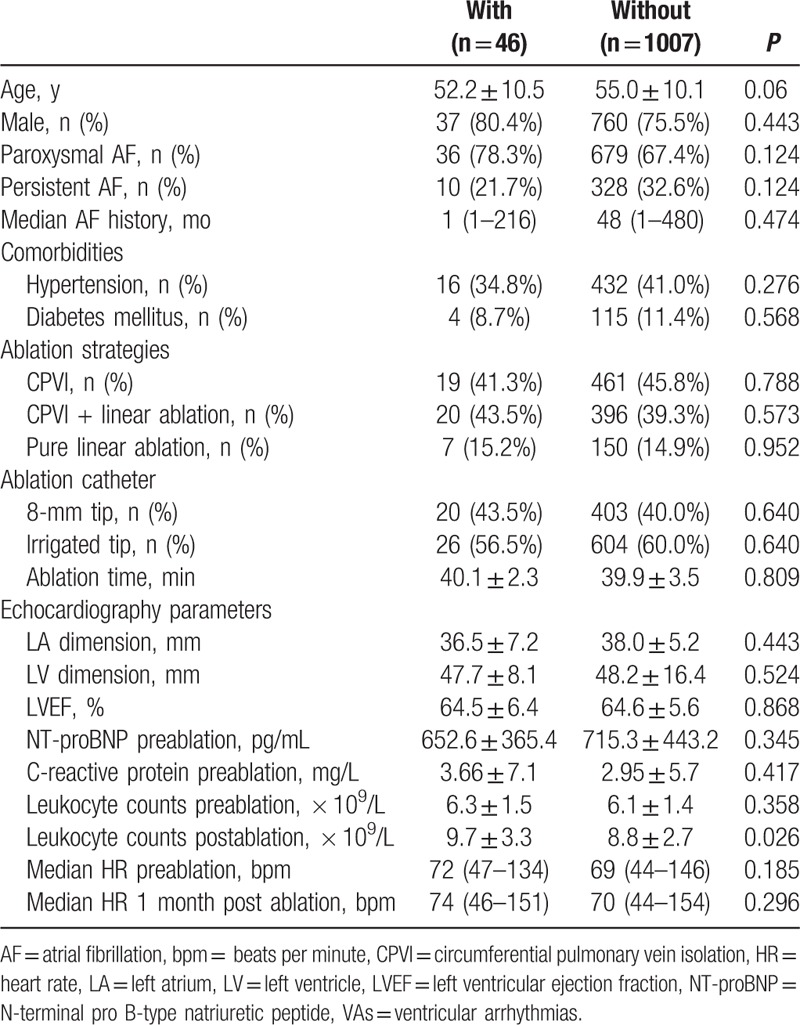
Comparison of characteristics of patients with and without new-onset VAs.

### 3.3 Characterization and prognosis of new-onset VAs

**Figure 1:**
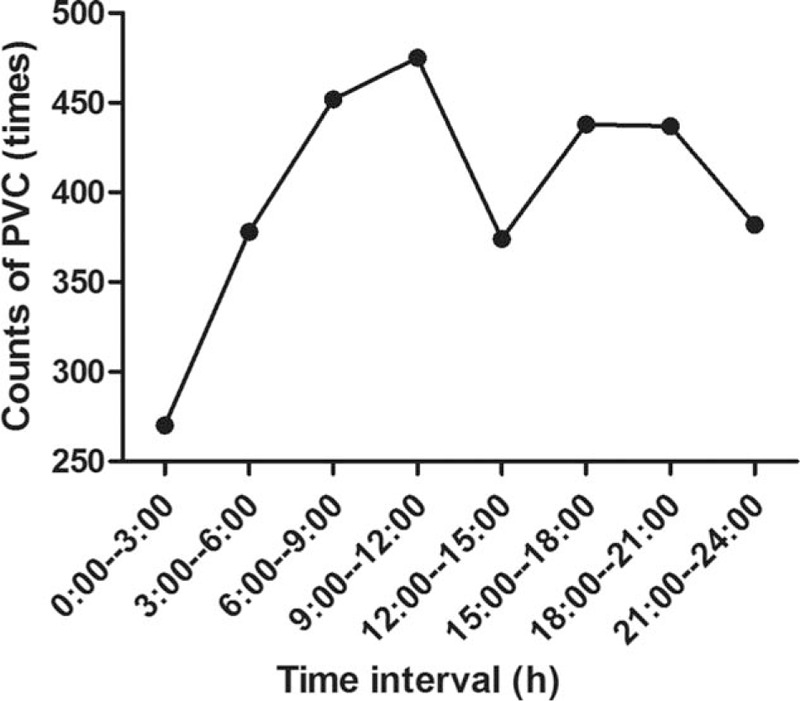
Comparison of origin of new-onset VAs post AF Ablation. AF = atrial fibrillation, LVNOT = left ventricular nonoutflow tract, LVOT = left ventricular outflow tract, RVNOT = right ventricular nonoutflow tract, RVOT = right ventricular outflow tract, VAs = ventricular arrhythmias.

**Figure 2:**
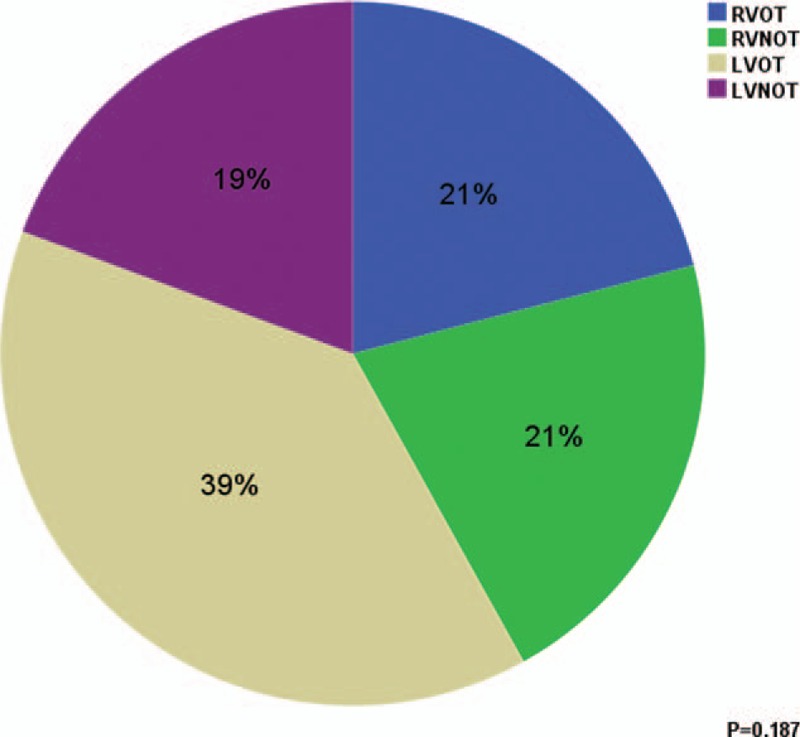
Circadian rhythm of new-onset PVC 1 month post AF ablation. AF = atrial fibrillation, PVC = premature ventricular contraction.

There were 31 patients who were asymptomatic (67.4%), the remaining 15 patients had palpitations associated with fullness in the chest. No patients had presyncope or syncope. The median number of PVC was 3161 (1001–27,407)/24 h at 1 month post ablation. PVC ≥10,000/24 h was found in 6 and PVC ≥5000/24 h in 14 patients. VAs originated from OT were recorded in 35 (76.1%) patients. Significant circadian rhythm of new-onset VAs was found (Fig. 1). More PVCs occurred in the daytime than in night, and each peak was found during the morning and afternoon. Comparison of the origin of VAs is shown in Figure 2, no significant statistical difference was found (*P* = 0.187).

**Figure 3:**
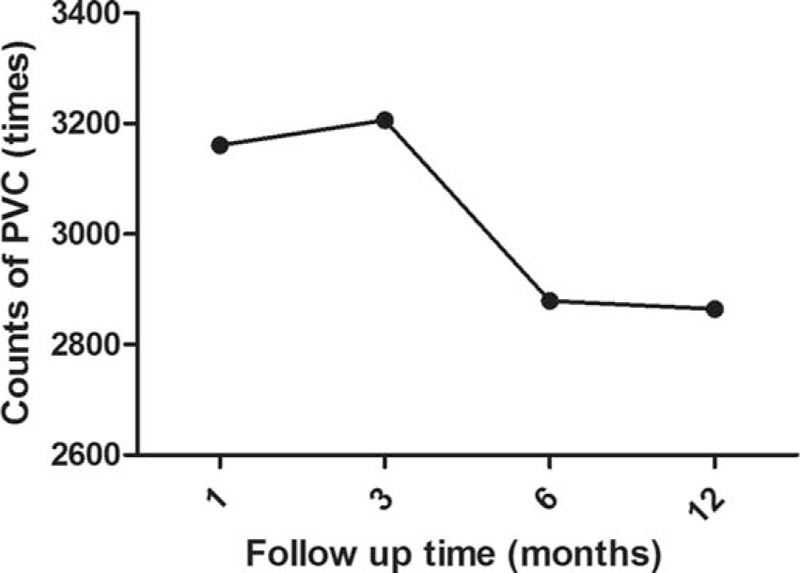
Comparison of median counts of new-onset PVC post AF ablation during follow-up. AF = atrial fibrillation, PVC = premature ventricular contraction.

There were 9 VAs which disappeared without any treatment in 6 patients within 6 months (13.0%), amongst which 6 originated from OT. The counts of new-onset PVC during different periods are compared in Figure 3. Three patients with symptomatic PVC ≥10,000/24 h underwent successful RFCA. No adverse cardiac event was recorded in patients with new-onset VAs including sustained VT, ventricular fibrillation, left ventricle (LV) enlargement, left ventricular ejection fraction (LVEF) decrease, and sudden cardiac death.

## 4 Discussion

This study is the first to describe the prevalence, characteristics and potential mechanism of new-onset VAs in a large cohort. The results enriched the knowledge of complications of AF ablation. Although extensive ablation strategies for AF may decrease the rate of AF recurrence, it may lead to more complications. Thus, extensive ablation strategies for AF may be used with caution. To our knowledge, only one study with a small sample size by Patel et al^[6]^ reported new-onset PVC post AF ablation. They studied 53 consecutive patients undergoing wide-area CPVI ablation, and found that the incidence of new-onset PVC was 11%. The relative low incidence of new-onset VAs found in our study may be attributed to more stringent inclusion criteria or the larger sample size.

The mechanism of new-onset VAs post AF ablation remains to be understood. With present understanding, 2 hypotheses have been proposed to explain this. First, drug-related proarrhythmia is a possible mechanism.^[12]^ However, previously ineffective antiarrhythmic drugs without demonstrated proarrhythmic effects were prescribed post ablation in our study, which is against this possibility. Second, Patel et al^[6]^ hypothesized that the modulation of adjacent autonomic ganglia by AF ablation developed new-onset OT PVC. They considered that increased mean HR post ablation reflected modulation of adjacent autonomic ganglia, and were associated with new-onset VAs. The results from our study did not support this point. However, it remains difficult to evaluate accurately remodeling of the cardiac autonomic nervous system. More evidence is needed to establish the possible mechanisms of cross talk between atrium and ventricle, post AF ablation. In addition, 40% of new-onset VAs in our study were of non-OT origin, which do not readily correlate with atrial autonomic ganglia.

Our findings prompted us to propose a new hypothesis that post-ablation inflammation may be an underlying mechanism. Extensive lesions in the atria caused by RFCA could induce an important inflammatory response. Previous studies have shown that early recurrences of AF are related to inflammatory response, and that the use of anti-inflammatory drugs including corticosteroids or colchicine could decrease systemic inflammation and improve the outcome of the procedure.^[13–15]^ Inflammatory response following myocardial infarction or myocarditis is related to the risk of new-onset VAs.^[16–18]^ Furthermore, as a traditional biomarker, serum leukocyte counts can be used as a marker of systemic inflammation.^[19–21]^ The results of this study showed that post-ablation increases in serum leukocyte counts ≥50% were associated with new-onset VAs. This result indicates that inflammatory response post RFCA may be one of the mechanisms responsible for new-onset VAs. Further studies are required to test our hypothesis.

It is worth mentioning that 9 VAs disappeared without any treatment in 6 patients within 6 months. Similar phenomenon can be observed in myocarditis. One possible explanation might be that inflammatory injury was transient.

MicroRNAs have been used as myocardial fibrotic and electrical alterations biomarkers in recent years. Sardu et al reported that microRNA expression might change after AF ablation, and the catheter ablation response may be upgraded by microRNA therapy to prevent cardiac electrical and fibrotic remodeling post ablation.^[22]^ However, whether the changes of microRNA expression post ablation are associated with new-onset VAs remains unclear, which should be clarified in future studies. Metabolic and oxidative stresses are associated with the generation and development of VAs. The mechanism may be that the metabolic and oxidative stresses induce ion channel changes in cardiac myocytes.^[23,24]^ Autonomic dysfunction is associated with AF and the ablation response.^[25]^ However, the quantitative analysis of autonomic dysfunction is relatively difficult. HR, HR variability, and deceleration capacity are often used to evaluate the autonomic dysfunction. In this study, we used HR to assess the autonomic dysfunction and no significant difference was found between patient with and without new-onset VAs.

## 5 Limitations

There are several limitations in our study. First, given the potential temporal variability in VAs burden in individuals, it is possible that only a 24-h Holter and 48-h of monitoring before ablation might have underestimated the prevalence of VAs. Second, although all patients underwent clinical follow-up including electrocardiograms and 24-h Holter monitor at prescribed intervals, asymptomatic episodes of VAs might have been missed, more rigorous follow-up methods may show our results to have underestimated the numbers of VAs. In addition, other metrics of inflammatory response were not used, for example the C-reactive protein and interleukin-6 post ablation. If other metrics displayed similar trends, this would add support for our hypothesis.

## 6 Conclusions

This study revealed a noticeable prevalence but relatively benign prognosis of new-onset VAs post RFCA for AF. Increased serum leukocyte counts ≥50% post ablation appeared to be associated with new-onset VAs, implying that inflammatory response caused by the ablation may be the underlying mechanism.
